# Distinct epidemiological profiles associated with inflammatory breast cancer (IBC): A comprehensive analysis of the IBC registry at The University of Texas MD Anderson Cancer Center

**DOI:** 10.1371/journal.pone.0204372

**Published:** 2018-09-24

**Authors:** Tamer M. Fouad, Naoto T. Ueno, Robert K. Yu, Joe E. Ensor, Ricardo H. Alvarez, Savitri Krishnamurthy, Anthony Lucci, James M. Reuben, Wei Yang, Jie S. Willey, Vicente Valero, Melissa L. Bondy, Massimo Cristofinalli, Sanjay Shete, Wendy A. Woodward, Randa El-Zein

**Affiliations:** 1 Department of Breast Medical Oncology, The University of Texas MD Anderson Cancer Center, Houston, TX, United States of America; 2 Morgan Welch Inflammatory Breast Cancer Research Program and Clinic, The University of Texas MD Anderson Cancer Center, Houston, TX, United States of America; 3 Department of Biostatistics, The University of Texas MD Anderson Cancer Center, Houston, TX, United States of America; 4 Department of Pathology, The University of Texas MD Anderson Cancer Center, Houston, TX, United States of America; 5 Department of Surgical Oncology, The University of Texas MD Anderson Cancer Center, Houston, TX, United States of America; 6 Department of Hematopathology, The University of Texas MD Anderson Cancer Center, Houston, TX, United States of America; 7 Department of Diagnostic Radiology, The University of Texas MD Anderson Cancer Center, Houston, TX, United States of America; 8 Department of Radiation Oncology, The University of Texas MD Anderson Cancer Center, Houston, TX, United States of America; 9 Department of Epidemiology, The University of Texas MD Anderson Cancer Center, Houston, TX, United States of America; Ohio State University Wexner Medical Center, UNITED STATES

## Abstract

**Background:**

To date, studies on inflammatory breast cancer (IBC) lack comprehensive epidemiological data. We analyzed detailed prospectively collected clinical and epidemiological data from the IBC Registry at The University of Texas MD Anderson Cancer Center.

**Methods:**

Patients with IBC (n = 248) were consecutively diagnosed and prospectively enrolled between November 2006 and April 2013. All patients were newly diagnosed and at least 18 years old. Secondary IBC was excluded. Overall 160 variables were collected and evaluated including sociodemographics, anthropometrics, tobacco and alcohol consumption, reproductive variables, and family history data.

**Results:**

Mean age at diagnosis was 51.6 (±11.5 SD) years, and the majority of patients were White (77.8%). A mean BMI ≥ 25 kg/m^2^, irrespective of menopausal status, was observed in 80.2% of all patients, with 82.6% of African Americans being obese. Approximately 42.2% of patients were ever smokers, and 91% reported ever being pregnant. A history of breastfeeding was reported in 54% of patients, with significant differences between ethnic groups in favor of White women (*P*<0.0001). Other reproductive factors such as use of birth control pills & hormone replacement therapy were also more frequently associated with White women compare to other ethnic groups (P < 0.05). In the multivariate Cox proportional hazard analysis, African American or Hispanic ethnicity, not having breastfed, higher clinical stage, and TNBC subtype were associated with shorter survival.

**Conclusion:**

Our data suggest that IBC is associated with distinct epidemiological profiles. This information could assist in targeting patients with specific preventive strategies based on their modifiable behavioral patterns.

## Introduction

Inflammatory breast cancer (IBC) is a rare, aggressive form of breast cancer that accounts for 1–3% of breast cancers diagnosed annually in the United States [1, 2]. The disease is characterized by the rapid appearance of redness, edema and peau d’orange skin due to occlusion of breast dermal lymphatics by tumor emboli [3,4]. Although patients with IBC progress rapidly and have a poor overall outcome, the introduction of multimodality therapy as standard of care improved the 5 year overall survival rates to up to 61% [5]. To date, epidemiological studies have shown that IBC presents at a younger age than non-IBC and that incidence is higher in African Americans than in Whites [2, 6–8]. Additionally, a high body mass index (BMI) has consistently been reported as a risk factor for IBC irrespective of menopausal status [9]. However, our ability to conduct a comprehensive study of the epidemiology of IBC has been hampered by the relative rarity of the disease and lack of consistency across IBC databases and registries [3,10]. In addition, large national registries lack details required to understand the epidemiological factors associated with disease development [2–11]. To address this issue, the Morgan Welch Inflammatory Breast Cancer Research Program and Clinic at The University of Texas MD Anderson Cancer Center developed an IBC registry in 2007. The registry objective is to prospectively collect clinical, epidemiological, and imaging data from patients with IBC.

Here we describe the findings of the IBC registry with regard to the epidemiological characteristics associated with diagnosis of IBC as well as their relationship to well known IBC risk factors: specifically, age, ethnicity, and BMI. Identifying risk modifiers could allow the classification of patients with IBC into subgroups with distinct epidemiological and/or behavioral patterns, leading to the development of better preventive strategies.

### Patients and methods

**Fig 1:**
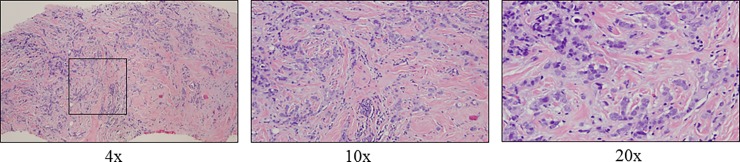
Hematoxylin and eosin stained tissue section of a breast core biopsy collected in the registry (x4, x10, x20). The biopsy shows invasive ductal carcinoma including loose clusters of tumor cells distributed in the stroma.

Patients were diagnosed between November 2006 and April 2013 by a multidisciplinary team of clinicians. Biopsy specimens [that included a standard biopsy for diagnostic purposes as well as a core biopsy, punch skin biopsy, lymph node biopsy and locoregional and/or distant metastasis biopsies] were reviewed by a breast pathologist. A clinical diagnosis of IBC was defined according to the criteria outlined in the 7^th^ edition of the *AJCC Cancer Staging Manual* and the recommendations of the international expert panel on IBC [5,12]. These two criteria overlap and were used to enroll patients in the IBC Registry at MD Anderson over the duration of this study. The standardized criteria required to establish a diagnosis of IBC are as follows: 1) Erythema and edema occupying at least one-third of the breast; 2) A rapid onset and duration of history of no more than 6 months (to differentiate IBC from long standing locally advanced breast cancer); 3) A core biopsy is essential to establish the presence of invasive carcinoma; 4) At least two skin punch biopsies are strongly recommended to detect dermal lymphatic invasion which according to the AJCC can be used to confirm the diagnosis but is neither required nor sufficient to make the diagnosis. Fig 1 shows a representative hematoxylin and eosin stained tissue section of a breast core biopsy collected in the registry (at 4x, 10x, 20x magnification).

Patients had to be at least 18 years old female and presenting with a new diagnosis of IBC. Both newly diagnosed patients who received multidisciplinary therapy at MD Anderson (Cohort 1) and newly diagnosed patients who received part of their treatment in the form of pre-surgical systemic therapy prior to coming to MD Anderson (Cohort 2) were enrolled into the study. Secondary IBC, defined as IBC that occurred after a history of non-IBC or IBC described as “secondary IBC” in the electronic medical record, was excluded. Patients with recurrent IBC or locally advanced breast cancer not meeting the criteria of diagnosis for IBC were also excluded from the analysis.

After obtaining a written informed consent, patients completed an epidemiological risk factor questionnaire with detailed sociodemographic characteristics, reproductive history, and family history. Data for analysis were obtained from the questionnaire, MD Anderson’s Patient History Database, and medical records. The variables of interest included age, ethnicity, marital status, tobacco and alcohol use, reproductive history, breast health (breast exams, mammograms), oral contraceptive and hormone replacement therapy (HRT) use, family history, and body mass index (BMI). Hormone receptor status was determined using immunohistochemistry (IHC) [13, 14]. HER2 status was considered positive if protein overexpression was classified as +3 on IHC staining or gene amplification was positive by fluorescent in situ hybridization (FISH). In addition, tissues were collected from all study participants. For Cohort 1, collection consisted of fresh frozen and formalin fixed and paraffin embedded tissue. Tissues were obtained from: Skin punch biopsies [up to 4 skin punch biopsies]; Tumor core biopsies [up to 4 Core biopsies]; Lymph node biopsies (if applicable); Locoregional and/or distant metastasis biopsies (if applicable); Mastectomy/Axillary dissection Tissue (if applicable); Paraffin blocks or up to 20 unstained slides per representative block (s) of primary breast cancer prior to primary systemic therapy if available were collected. For Cohort 2 participants, who had their initial biopsies outside the institution and have paraffin blocks or unstained slides available for tissue banking: Core biopsy, punch skin biopsy, lymph node biopsy or distant metastasis in paraffin blocks or up to 20 unstained slides per representative block(s) were collected, if applicable.

Outcome measures evaluated for this analysis were pathological complete response (pCR) and overall survival. pCR was defined as absence of invasive residual carcinoma (pT0, pN0) after completion of neoadjuvant treatment. Overall survival was defined as the period from date of diagnosis to the date of death or to last follow-up (records were assessed on 06/2015). The study was approved by the Institutional Review Board at The University of Texas MD Anderson Cancer Center.

### Statistical methods

Statistical analyses were conducted using SAS for Windows (version 9.4). Standard descriptive statistics and frequency tabulation by using SAS’s PROC MEANS and PROC FREQ functions were used to summarize patient characteristics. All variables that were significant in univariate analysis and variables of interest were entered into a multivariate model. A Cox proportional hazard model was used to calculate hazard ratios and 95% confidence intervals. A *P-*value of less than 0.05 was considered significant.

## Results

### Study participants

Two-hundred sixty-five patients were identified through the IBC registry. Seventeen patients were excluded due to: a diagnosis of secondary IBC (n = 8), recurrent IBC (n = 3), locally advanced breast cancer (n = 1), drop-off the study or refusal to participate in the study (n = 5). A total of 248 patients were included in the final analysis. Cohort 1 patients comprised 73.4% (n = 182) of the study population, while cohort 2 patients comprised the remaining 26.6% (n = 66).

### Patient characteristics

Demographic and clinical characteristics for all study participants are presented in Table 1. The mean age at diagnosis ± standard deviation (SD) was 51.6 ± 11.5 years (range, 23–80.2 years). White patients comprised 77.8% of the population, 9.3% were African Americans, 10.5% were Hispanic, and 2.4% were Asian/Asian-Pacific. Due to the small number of Asians, they were excluded from any further analyses. Sixty-three percent of patients presented with stage III disease, while 36.8% had stage IV disease. The hormone receptor (HR)-positive subtype (positive for estrogen receptor [ER] and/or progesterone receptor [PR]) was present in 52.8%, while HER2-positive and triple-negative (TNBC) subtypes were present in 38.7% and 25.4% of patients, respectively. The demographic and clinical characteristics for the study participants by cohort are presented in S1 Table.

**Table 1:** Demographic and clinical characteristics for the study population as a whole and by racial/ethnic group^a^.

Variables	% (No. of Patients)^b^				
	Overall	White	African American	Hispanic	Exact *P*-value
	N = 248	N = 193	N = 23	N = 26	
**Age at Diagnosis, y**, Mean ± SD	51.6 ±11.5	52.7 ± 10.5	50.3 ± 15.7	46.7 ± 13.4	0.3882
**BMI at Diagnosis,** kg/m^2^ Mean ± SD	30.9 ± 7.9	30.6 ± 7.5	37.2 ± 10.7	28.7 ± 5.8	0.7593
**BMI Category**					**0.0059**
Normal	19.7(49)	19.7 (38)	4.4 (1)	23.1 (6)	
Overweight	30.6 (76)	32.1 (62)	13.0 (3)	38.5 (10)	
Obese	49.5 (123)	48.2 (93)	82.6 (19)	38.5 (10)	
**Smoking**					**0.0047**
Ever	42.2 (103)	47.9 (91)	30.4 (7)	20.0 (5)	
Never	57.8 (141)	52.1 (99)	69.6 (16)	80.0 (20)	
**No. of cigarettes/day** ± SD	16.3 ± 10.8	16.5 ± 11.0	22.2 ± 6.6	7.0 ± 3.0	**0.0486**
**Childhood Exposure to Secondhand Smoke**					**0.0036**
Never	35.4 (70)	32.3 (51)	25.0 (5)	70.6 (12)	
Sometimes	13.1 (26)	11.4 (18)	25.0 (5)	11.8 (2)	
Regularly	51.5 (102)	56.3 (89)	50.0 (10)	17.6 (3)	
**Pregnancy**					0.1165
Ever	91.1 (225)	92.7 (178)	87.0 (20)	88.5 (23)	
Never	8.9 (22)	7.3 (14)	13.0 (3)	11.5 (3)	
**No. of Pregnancies** ± SD	2.7 ± 1.5	2.5 ± 1.2	3.8 ± 2.3	3.0 ± 1.8	**0.0006**^c^
**Age at First Pregnancy** n = SD [Range]	23.4 ± 5.4 [14–46]	23.8 ± 5.4 [15–46]	19.4 ± 4.6 [14–29]	23.2 ± 5.5 [15–36]	**<0.0001**^c^
**Breastfeeding**					**<0.0001**
No	46.0 (92)	40.3 (64)	89.5 (17)	57.9 (11)	**<0.0001**
Yes	54.0 (108)	59.8 (95)	10.5 (2)	42.1 (8)	
**Menopause**					**0.0074**
No	31.0 (76)	26.0 (50)	43.5 (10)	53.9 (14)	
Yes	69.0 (169)	74.0 (142)	56.5 (13)	46.2 (12)	
**Hormone Replacement**					**0.0231**
No	72.2 (169)	67.4 (122)	91.3 (21)	88.0 (22)	
Yes	27.8 (65)	32.6 (59)	8.7 (2)	12.0 (3)	
**Prior Cancer History**					0.0839
No	90.2 (211)	87.2 (156)	100 (23)	100 (26)	
Yes	9.8 (23)	12.8 (23)	0 (0)	0 (0)	
**Clinical Stage**					**0.0356**
IIIB	33.6 (83)	37.5 (72)	8.7 (2)	30.8 (8)	
IIIC	29.6 (73)	26.0 (50)	43.5 (10)	42.3 (11)	
IV	36.8 (91)	36.5 (70)	47.8 (11)	26.9 (7)	
**Hormone Receptor Status**					**0.0110**
HR+/HER2-	35.9 (89)	38.3 (74)	43.5 (10)	15.4 (4)	
HR-/HER2+	21.8 (54)	21.2 (41)	4.4 (1)	34.6 (9)	
HR+/HER2+	16.9 (42)	18.1 (35)	8.7 (2)	15.4 (4)	
HR-/HER2-	25.4 (63)	22.3 (43)	43.5 (10)	34.6 (9)	
**pCR Rate**					**0.0107**
NO	78.4 (123)	81.1 (103)	90.9 (10)	56.3 (9)	
YES	21.7 (34)	18.9 (24)	9.1 (1)	43.7 (7)	

^a^ Asian/Asian-Pacific patients are not shown because of small sample size (n = 6).

^b^ Data represent percentage and number of patients unless otherwise indicated.

^c^ Chi-square test.

Overall mean BMI was 30.9 kg/m^2^, with only 19.7% of patients having a BMI ≤ 24.9 kg/m^2^, while 49.5% were obese (BMI > 30 kg/m^2^). Approximately 56.5% of obese patients were categorized as class I (BMI 30–34.9 kg/m^2^), 26.2% were class II (BMI 35–39.9 kg/m^2^), and 17.2% class III (BMI > 40 kg/m^2^). Cohort 1 patients were older than cohort 2 patients (mean age ± SD, 53.0 ± 11.4 years vs. 48.0 ± 11.2; *P =* 0.8), had a higher mean BMI with an over-representation of the obese category, and were diagnosed at a later stage of disease.

The majority of patients (57.8%) were never-smokers. Among the ever-smokers, the average number of years smoked was 20 years, with an average of 16.3 cigarettes/day. The mean age of smoking initiation was 19.7 years (range 8–47 years), and the mean age at cessation for those who quit was 37.2 years (range 10–72 years).

Upon comparing the demographic and clinical characteristics of the study participants by ethnicity (Table 1), we observed non-significant differences among the ethnic groups with respect to age (*P* = 0.388) and overall BMI (*P* = 0.759). However, the highest mean BMI was among African Americans (mean, 37.2 kg/m^2^), followed by Whites and Hispanics (30.6 kg/m^2^ and 28.7 kg/m^2^, respectively). Approximately 83% of African Americans were obese (BMI ≥ 30 kg/m^2^) compared with 48.2% and 38.5% of Whites and Hispanics, respectively (*P* = 0.0059). Among the obese African Americans, 37% were morbidly obese (BMI ≥ 40 kg/m^2^), compared to 14% and 10% of obese Whites and Hispanics, respectively.

A significantly higher frequency of ever-smokers was observed among White patients (47.9%) as compared to African Americans or Hispanics (30.4% and 20%, respectively; *P* = 0.0047). However, smoking intensity (cigarettes/day) was significantly higher among African Americans than among Whites (*P* = 0.04). Regular childhood exposure to secondhand smoke was reported in 51.5% of the patients and was significantly different among ethnic groups (*P* = 0.004), with the lowest exposure among Hispanics. Alcohol consumption was reported among 79% of the population, with the highest frequency among Whites (82%) and the lowest among Hispanics (58%; *P* <0.0001).

While Whites and Hispanic patients were almost equally represented in stage III disease (64% and 73%, respectively), African Americans were under-represented in stage III disease (52%) and over-represented in stage IV disease (48%, vs. 36% and 25% for Whites and Hispanics, respectively; *P* = 0.0356). HR status and HER2 status were significantly different among ethnic groups (*P* = 0.0110). HR^+^ (ER^+^/PR^+^)/HER2^-^ was observed in 35.9% of patients, followed by TNBC (HR^-^/HER2^-^) (24.5%), HR^-^/HER2^+^ (21.8%), and HR^+^/HER2^+^ (16.9%). African Americans had the highest frequency of triple-negative disease (43.5%), followed by Hispanics (34.6%) and Whites (22.3%). White patients had the highest frequency of HR^+^/HER2^+^ at 18.1%, and Hispanic patients had the highest frequency of HER2^+^ (50%).

### Breast symptoms and lag time to seeking medical advice

Self-discovery through breast exam was reported in 84% of patients. The majority of patients (79%) reported having annual mammograms. Redness was reported by 62.1% of patients, edema/fullness, 48%; skin dimpling/discoloration, 46%; a lump, 26%; and nipple inversion, 16%. Factors significantly associated with redness were age at diagnosis (odds ratio [OR] = 0.968, 95%CI = 0.944–0.993; *P* = 0.0117); edema (OR = 0.355, 95%CI = 0.2–0.629; *P* = 0.0004); and presence of a lump (OR = 2.613, 95%CI = 1.374–4.97; *P* = 0.0034). Among White and Hispanic patients, the main breast change was redness, while growth/fullness were the main changes in African Americans.

A breast lump was reported in only 26% of cases, mainly among 39%, 42%, and 22% of African American, Hispanics, and Whites, respectively (*P* = 0.007). Thirty-six percent of patients with a lump were diagnosed with stage IIIC disease, as compared to 18.5% and 24.4% with stage IIIB and stage IV, respectively (*P* = 0.049). The majority of patients presenting with a lump had the TNBC subtype (36.5%; *P* = 0.038).

The average time lag between noticing breast changes to seeking medical advice was 46 days (median 21 days; range 0–450 days). The longest delay was among Whites (average 49.9 days), followed by African Americans (37.1 days) and Hispanics (33.8 days). The top reason for the delay was believing the symptoms were not important (47% of patients). Other reasons [in 53% of patients] were: fear, breastfeeding, no time to visit a clinic or lack of insurance. A physician diagnosis of breast cancer was made in 38% of patients, while an initial diagnosis of IBC was made in only 4% and further testing was recommended for 20% of the cases. Patients reported being initially treated with antibiotics in 38% of cases, while 24% reported being misdiagnosed as having breastfeeding-related changes, an allergic reaction, insect bites or cysts.

### Reproductive variables

The mean age at menarche was 12.6 years (range, 8–17 years). One hundred and sixty nine patients were postmenopausal, with 44% reporting natural menopause, 34% reporting artificial menopause due to hysterectomy and 20% due to chemotherapy. Hormone Replacement Therapy [HRT] use was reported in 72% of patients (range, 1 month—29 years). In addition, 74.4% of patients reported using birth control, median duration 7 years (range, 3 months—25 years). History of birth control/HRT use was highest among Whites (*P* = 0.005 and *P* = 0.023).

A history of ever being pregnant was reported in 91% of patients. Overall mean age at first birth of 23.4 ± 5.4 years. Among African Americans, the mean age was 19.4 ± 4.6 years and significantly different from Whites and Hispanics (23.8 ± 5.4 and 23.2 ± 5.5 years, respectively; *P* <0.0001).

**Fig 2:**
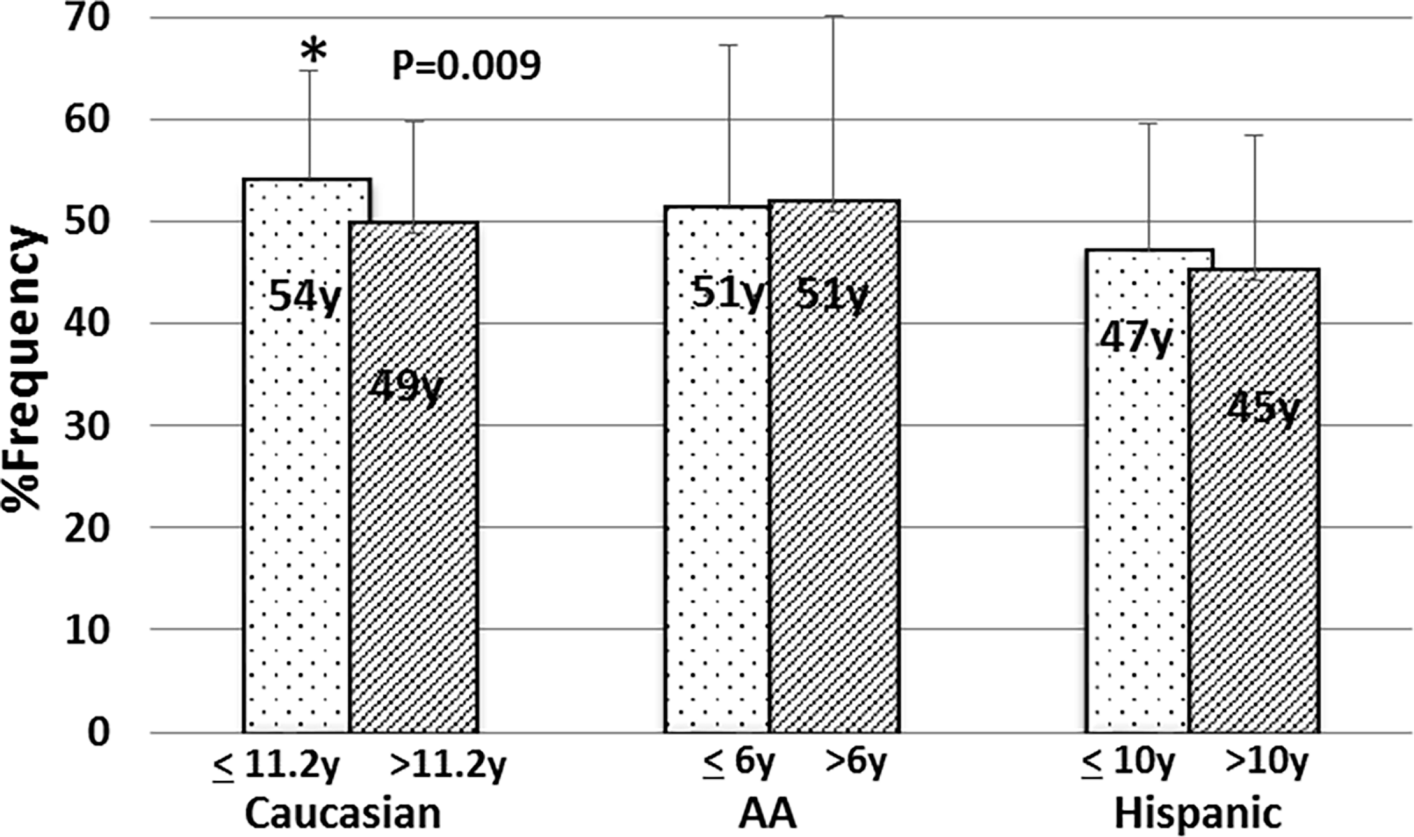
Time between age at menarche and age at first birth in association with age at diagnosis by ethnic group. For each group, the mean time between menarche and first birth was used to divide the group, and the mean age at IBC diagnosis for each subgroup was determined.

The overall mean number of pregnancies was 2.7 (range, 1–10) which was significantly higher for African Americans (3.8, range 1–10) than Whites and Hispanics (2.5, range, 1–7; and 3.0, range 1–9; *P* = 0.0006). The average time between pregnancies was 4.2 years (1–13 years).The mean duration between age at menarche and age at first birth was 10.7 ± 5.7 years. The longest duration was among Whites, 11.2 ± 5.5 years, compared to 10.2 ± 6.4 years for Hispanics (*P* = 0.043) and 6.5 ± 5.1 years for African Americans (*P* = 0.0003). Interestingly, the longer duration between menarche and first birth was significantly associated with younger age at diagnosis among Whites. The mean age at diagnosis for patients with a mean duration ≤ 11.2 years was 54.2 ± 10.7 years, vs. 49.9 ± 9.9 for those with a duration > 11.2 years (*P* = 0.009). This association was not seen in Hispanic or African American patients (Fig 2). A history of breastfeeding was reported in 54.0% of patients, with only 10.5% of African Americans ever breastfeeding vs. 59.8% of Whites and 42.1% of Hispanics (*P* <0.0001).

### Family history of breast cancer and/or gynecologic malignancy

A family history of breast cancer in a first-degree relative (mother, sister, or daughter) was reported in 13% of patients. A family history of breast and/or gynecologic malignancy was present in 21% of patients and was associated with a history of previous cancers [mainly non-melanoma skin cancer]. A history of previous cancers was exclusively seen among Whites (13% vs. 0% in African Americans and Hispanics; *P* = 0.022).

### Pathological complete response rate

The pCR rate in stage III IBC patients (n = 138) was 21.7%. When stratified by ethnicity, the lowest pCR rate was associated with African American ethnicity (*P* = 0.0107). Stratified by tumor subtype, pCR was significantly associated with the HER2^+^ subtype (*P* = 0.0004).

### Survival analysis

The median overall survival was 97.08 months (95%CI = 84.96–113.4); the mean overall survival was 77.6 months (standard error of the mean [SEM] = ± 3.3 months). Age at diagnosis did not significantly affect survival in the overall cohort (*P* = 0.2114). In multivariate Cox proportional hazard analysis, African American or Hispanic ethnicity, not having breastfed, higher clinical stage, and TNBC subtype were associated with shorter survival (Table 2). Age at diagnosis, number of pregnancies, menopause status, BMI, smoking status, age of menarche, and HRT or oral contraceptive use were not significantly associated with overall survival.

**Table 2:** Multivariate analysis of association of the risk of developing IBC with 11 covariates.

Variables	*P*-value	Hazard Ratio	95% Confidence Limits	
**Age at Diagnosis**	0.3096	0.986		(0.986, 0.959)
**Ethnicity**				
African American vs White	**0.0227**	**2.553**		**(2.553, 1.14)**
Hispanic vs White	**0.0136**	**3.537**		**(3.537, 1.297)**
**Number of Pregnancies**	0.3626	1.089		(1.089, 0.907)
**Menopause Status**	0.2509	1.62		(1.62, 0.711)
**BMI**	0.0732	0.959		(0.959, 0.917)
**Smoking Status**				
Former vs Never	0.2423	1.432		(1.432, 0.784)
Current vs Never	0.1223	2.13		(2.13, 0.816)
**Age of Menarche**	0.2483	0.894		(0.894, 0.74)
**Clinical Stage**				
IIIC vs IIIB	**0.0162**	**2.439**		**(2.439, 1.179)**
IV vs IIIB	**0.0019**	**3.023**		**(3.023, 1.504)**
**Breastfeeding**				
Yes vs No	**0.0151**	**0.455**		**(0.455, 0.241)**
**Hormone Replacement Therapy**	0.2955	1.419		(1.419, 0.737)
**Subtype**				
HR-/HER2+ vs HR-/HER2-	**0.0023**	0.232		(0.232, 0.091)
HR+/HER2- vs HR-/HER2-	0.1244	0.603		(0.603, 0.316)
HR+/HER2+ vs HR-/HER2-	**0.0055**	0.215		(0.215, 0.073)

The median overall survival among stage III patients was 113.4 months (CI = 97.1–113.4) with a mean ± SEM of 84.1 ± 4.0 months. For stage IV patients, the median was 56.7 months (CI = 39.7–NA), and the mean ± SEM was 53.9 ± 3.9 months. Results for survival by subtype are reported in mean days/years since our population breakdown did not allow for stable reporting using the medians. The overall survival time was shortest for HR^-^/HER2^-^ subtype (54.6 ± 6.5 months), followed by HR^+^/HER2^+^ (59.4 ± 2.6 months), HR^+^/HER2^-^ (61.1 ± 3.4 months), and HR^-^/HER2^+^ (76.1 ± 3.6 months). The overall survival time was shorter for patients with no history of breastfeeding (mean ± SEM = 61.0 ± 5.4 months, ~5.08 years) than for patients that breastfed (mean ± SEM = 73.6 ± 2.7 months, ~ 6.13 years). By multivariate analysis (Table 2), a history of breastfeeding was associated with better survival independent of clinical stage, disease subtype, or number of pregnancies (HR = 0.455; 95% CI: 0.46–0.24). Survival time by breastfeeding status was also assessed by ethnicity and was found to be longer among White patients who reported breastfeeding (mean ± SE = 75.2 ± 2.7 months) than among Hispanics who reported breastfeeding (mean ± SE = 38.2 ± 3.9 months); survival among the African Americans was not assessed due to lack of breastfeeding.

## Discussion

To our knowledge, this study is one of the largest analysis of a prospectively collected epidemiological dataset for IBC at a single institution. The study design allowed us to comprehensively document and assess the impact of various epidemiological factors in addition to tumor and patient characteristics. We identified epidemiological profiles that were associated with distinct behavioral patterns among ethnic groups with regard to reproductive history, breastfeeding, smoking, and obesity (Table 3).

**Table 3:** Descriptive classification of epidemiological factors.

Profile A	Profile B
• Ethnicity: White • Age: 5^th^ decade (Median = 53 years) • Smoking (*P =* 0.04) • Reproductive factors: post-menopausal, breast feeding, birth control pills & hormone replacement therapy (*P* < 0.05)	• Ethnicity: Black • Age: 4^th^ decade (Median = 43 years) • BMI (83% Class III obesity) (*P =* 0.006)

Our results suggest a significant association between pregnancy, lactation, and the risk of developing IBC, particularly among different ethnicities. Bonnier et al previously reported a high number of IBC cases in patients presenting with pregnancy-related breast cancer compared to non-pregnant patients with breast cancer (26% vs. 9.1%, *P* < .00001) [15]. Similarly, in a Tunisian study, higher rates of IBC were seen in women who were currently pregnant or lactating at the time of diagnosis than in patients who were not pregnant (79% vs. 67%, *P* = .01) [16]. Moreover, we have recently reported that certain risk factors such as age at first birth (≥ 26 years), breastfeeding, and smoking may be associated with specific IBC subtypes [17]. Women with a history of breastfeeding had a lower risk of developing triple-negative and luminal IBC [17].

**Fig 3:**
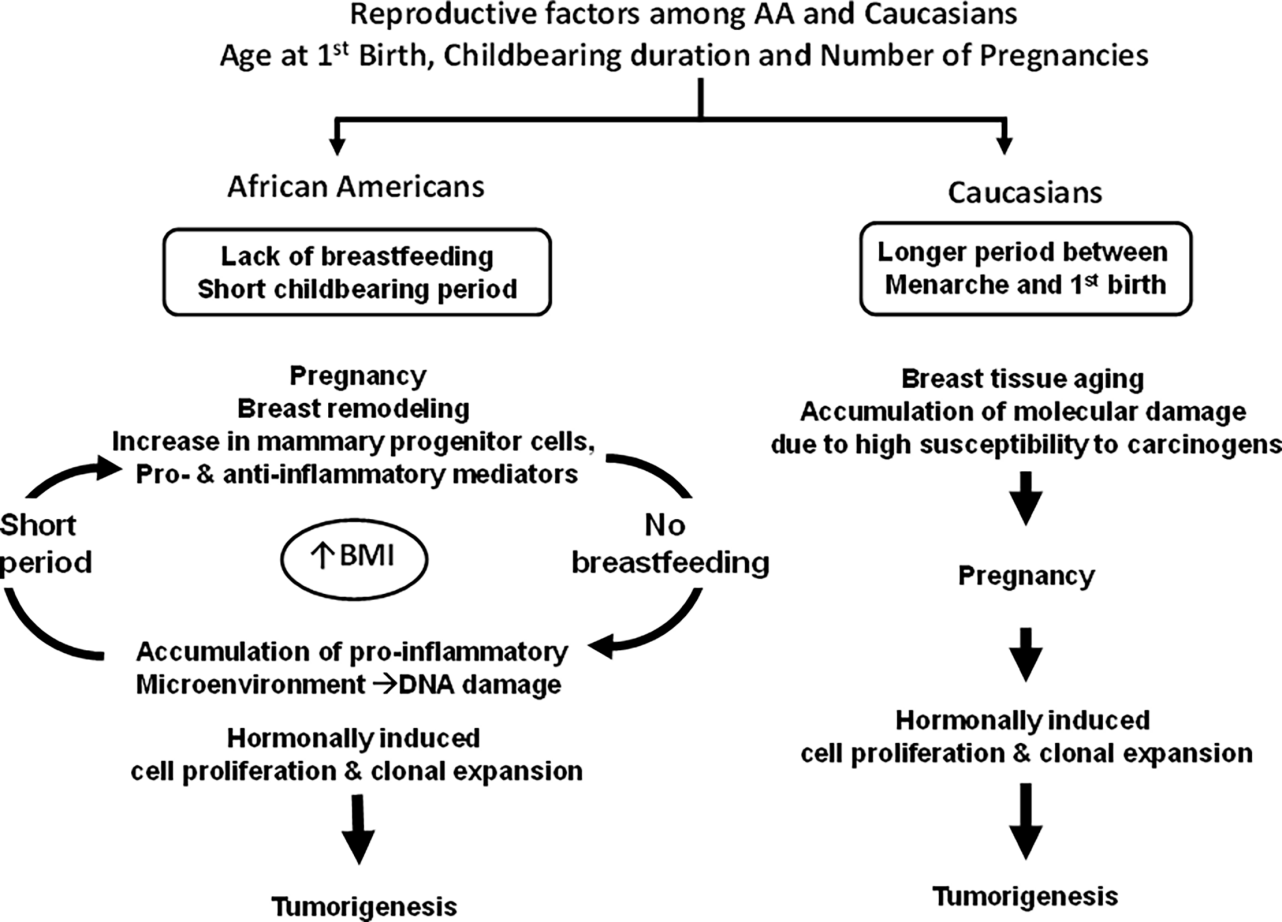
Proposed scenarios for the role of reproductive factors associated with IBC in our cohort.

Both younger age at first birth and multiparity were significantly represented in the African American patients. However, we speculate that shorter period between age at menarche and age at first birth and lack of breastfeeding among this population potentially creates a cancer-prone microenvironment in the breasts [18, 19]. During pregnancy, breast remodeling takes place with an increase in mammary progenitor cells and pro- and anti-inflammatory mediators, which should drop after weaning. However, lack of breastfeeding leads to accumulation of pro-inflammatory microenvironment and production of oxidative stress, leading to DNA damage [20]. This cycle is repeated with subsequent pregnancies, which not only leads to further enrichment of the tumorigenic microenvironment but also facilitates hormonal-induced cell proliferation and clonal expansion [21]. The scenario is different for Whites, for whom average older age at first birth and fewer number of pregnancies are reported risk factors for development of breast cancer. In addition, a longer period between age at menarche and age at first birth causes breast tissue aging, in which accumulation of molecular damage takes place due to high susceptibility of undifferentiated breast tissues to carcinogens [22]. During pregnancy, breast remodeling takes place as well as hormonal induction of cell proliferation, which in the presence of existing molecular damage, leads to clonal expansion and tumorigenesis [23] (Fig 3).

Chang et al were among the first to report the association between a high BMI and the development of IBC, which, in contrast to the association in non-IBC, is not limited to the postmenopausal subgroup [9, 24]. In addition to confirming this association, our results show that BMI varied significantly by ethnicity but not menopausal status (Table 1). Similar to the study by Chang et al, the majority of patients in our analysis were never-smokers, while the rate of smoking history was in line with reported national averages in the US [9].

White ethnicity was more frequently associated with a history of smoking as well as several reproductive risk factors such as postmenopausal status, breastfeeding, and oral contraceptive and HRT usage. Whites were also more likely to develop hormone-receptor-positive tumors. On the other hand, 83% of African Americans had class III obesity, and African American ethnicity was more frequently associated with triple-negative IBC.

The age frequency distribution curve in our analysis confirms previously reported age-specific incidence rates, with peak diagnosis during the fifth decade. This contrasts with non-IBC, which tends to peak 10 years later and is associated with a dip known as Clemmesen's hook that has been attributed to menopause [9, 10]. Due to our relatively small sample size, the differences in age distribution among different ethnicities did not reach statistical significance [2, 7,8,10]. Approximately 13% of women in our cohort reported having a first-degree female relative with breast cancer, in line with other reports for both IBC and non- IBC [9]. Patients with a positive family history of non-IBC, however, were found to have a similar incidence and age distribution as seen for non-IBC [23, 25].

From a clinical perspective, 84% of patients reported diagnosis through self-discovery, despite almost 80% having undergone screening mammography. This means of discovery can be attributed to to the frequent absence of a mass in IBC, but also due to its confusion with mastitis. Almost half the women in this study delayed seeking medical attention because they did not believe their symptoms were important. In our study, a breast lump was reported in less than a third of the patients and was least reported in Whites (*P* = 0.007), in whom erythema was the most commonly reported breast change. This is supported by radiological findings where the use of diagnostic MRI was only able to detect a single dominant mass in 38% of cases, while the majority of masses detected were multiple, small, and confluent [26]. Taken together, these data highlight the ineffectiveness of screening programs and the importance of awareness efforts in diagnosing this disease. In terms of outcome, our results suggest an association with certain risk factors may also predict worse outcome. In addition to clinical stage and tumor subtype, risk factors such as ethnicity (African American, Hispanic) and breastfeeding, but not high baseline BMI, were associated with survival.

The limitations of our study include the potential for bias in patient selection at a single tertiary referral institution, and lack of a control group. However, our results strongly suggest that there are distinct epidemiological characteristics and behavioral patterns associated with development and survival of IBC. Although different epidemiological risk factors can be drawn across ethnicities, the significance was somewhat limited by our small sample size. Currently, our ongoing IBC Registry Trial is open and accruing IBC patients (ClinicalTrials.gov NCT00477100).

The main strength of this study lies in the extensive amount of epidemiological data collected from patients upon enrollment. This allowed us to analyze over 160 epidemiological and clinical variables and identify several patient profiles that were distinct in terms of reproductive history, breastfeeding, smoking, and obesity. While by no means conclusive, this descriptive classification suggests that IBC could be linked to different modifiable risk patterns among ethnic groups. This could lead to a better understanding of the heterogeneous etiology of this disease, as well as provides a new paradigm in targeting patients with specific preventive strategies based on their modifiable behavioral patterns.

## Supporting information

S1 Table Demographic and clinical characteristics by study cohort. (DOCX)
