# Supplementary material for: Distinct epidemiological profiles associated with inflammatory breast cancer (IBC): A comprehensive analysis of the IBC registry at The University of Texas MD Anderson Cancer Center
Source: PLoS One. 2018 Sep 24;13(9):e0204372. doi: 10.1371/journal.pone.0204372 (PMC6152950; doi:10.1371/journal.pone.0204372)
Supplement: S1 Table — (DOCX) [file pone.0204372.s001.docx]

|  | **Overall Cohort (n=248)** | **Cohort 1**  **(n =182)** ^a^ | **Cohort 2**  **(n =66)** ^b^ | **Fisher’s Exact Test *p*-value** |
| --- | --- | --- | --- | --- |
|  |  |  |  |  |
| **Age at Diagnosis** (Mean ± SD) | 51.6 ± 11.5 | 53.0 ± 11.4 | 48.0 ±11.2 | 0.8469 |
| **Ethnicity** [% (n)] |  |  |  | 0.5851 |
| White | 77.8 (193) | 78.0 (142) | 77.3 (51) |  |
| Black | 9.3 (23) | 10.4 (19) | 6.1 ( 4) |  |
| Hispanic | 10.5 (26) | 9.3 (17) | 13.6 (9) |  |
| Asian/Asian Pacific | 2.4 (6) | 2.2 (4) | 3.0 (2) |  |
| **BMI at Diagnosis**  Kg/m^2^ (Mean ± SD) | 30.9 ± 7.9 | 31.4 ± 8.3 | 29.5 ± 6.6 | 0.9133 |
| **BMI Category** [ % (n)] |  |  |  | 0.0852 |
| Normal | 19.7 (49) | 16.5 (30) | 28.8 (19) |  |
| Over weight | 30.6 (76) | 30.8 (56) | 30.3 (20) |  |
| Obese | 49.6 (123) | 52.8(96) | 40.9 (27) |  |
| **Smoking Status** [% (n)] |  |  |  | 0.2360 |
| Ever | 42.2 (103) | 44.5 (81) | 35.5(22) |  |
| Never | 57.8 (141) | 55.5 (101) | 64.5 (40) |  |
| **Pregnancy** |  |  |  | 1.000 |
| Ever | 91.1 (225) | 90.6 (165) | 90.9 (60) |  |
| Never | 8.9 (22) | 8.7 (16) | 9.1 (6) |  |
| **Age at 1^st^ Pregnancy** (n=27 missing data)  Mean ± SD [Range] | 23.4 + 5.4  [14-46] | 23.0 + 5.4  [14-46] | 24.5+ 5.3  [15-36] | 0.1628* |
| **Breastfeeding (n=48 missing data)** |  |  |  | 0.4370 |
| No | 46.0 (92) | 47.8 (68) | 41.3 (24) |  |
| Yes | 54.0 (108) | 51.1 (74) | 58.6 (34) |  |
| **Menopause** |  |  |  | 1.000 |
| No | 31.0 (76) | 31.3 (56) | 30.3 (20) |  |
| Yes | 68.9 (169) | 68.7 (123) | 69.7 (46) |  |
| **Hormone Replacement (n=14 missing data)** |  |  |  | 0.4064 |
| No | 72.2 (169) | 70 (122) | 77 (47) |  |
| Yes | 27.7 (65) | 29.4 (51) | 22.9 (14) |  |
| **Clinical Stage** [% (n)] |  |  |  | **0.0022** |
| IIIB | 33.6 (83) | 27.1 (49) | 51.5 (34) |  |
| IIIC | 29.6 (73) | 32.6 (59) | 21.2 (14) |  |
| IV | 36.8 (91) | 40.3 (73) | 27.3 (18) |  |
| **Hormone status** [% (n)] |  |  |  | 0.9054 |
| HR+/ HER2- | 35.9 (89) | 36.3 (66) | 34.9 (23) |  |
| HR-/ HER2+ | 21.8 (54) | 22.0 (40) | 21.2 (14) |  |
| HR+/ HER2+ | 16.9 (42) | 17.6 (32) | 15.2 (10) |  |
| HR-/ HER2- | 25.4 (63) | 24.2 (44) | 28.8 (19) |  |
| **pCR rate** [% (n)] |  |  |  | **0.0083** |
| NO | 78.3 (123) | 73.3 (85) | 92.7 (38) |  |
| Yes | 21.7 (34) | 26.7 (31) | 7.3 (3) |  |

^a^ Cohort 1: newly diagnosed patients who received multidisciplinary therapy at MD Anderson
^b^ Cohort 2: newly diagnosed patients who received pre-surgical systemic therapy prior to arrival at MD Anderson.
